# IGFBP2 function as a novel biomarker for active lupus nephritis

**DOI:** 10.1007/s00109-022-02241-z

**Published:** 2022-08-25

**Authors:** Hui Li, Jun Liang, Yingying Gao, Min Liu, Nan Xia, Wei Kong, Lisha Zheng, Yikun Zhang, Zutong Li, Hongwei Chen, Shanshan Liu, Lingyun Sun

**Affiliations:** 1 Department of Rheumatology and Immunology, Drum Tower Clinical Medical College of Nanjing Medical University, Nanjing, Jiangsu 210008 China; 2 Northern Jiangsu People’s Hospital Affiliated to Clinical Medical College, Yangzhou University, Yangzhou, 225002 China; 3 Key Laboratory for Biomechanics and Mechanobiology of Ministry of Education, School of Biological Science and Medical Engineering, Beihang University, Beijing, China; 4 PLA Strategic Support Force Characteristic Medical Center, 9 Anxiang Beili, Chaoyang District, Beijing, China; 5 Department of Rheumatology and Immunology, The Affiliated Drum Tower Hospital, Nanjing University Medical School, Nanjing, Jiangsu 210008 China

**Keywords:** Lupus nephritis, IGFBP2, T cells, Renal pathology

## Abstract

**Abstract:**

In search for new targets for the diagnosis and treatment of lupus nephritis (LN), we employed TMT-liquid chromatography-triple quadrupole mass spectrometry (TMT-LC–MS/MS) combined with RNA-seq and identified a panel of proteins that was dysregulated both at protein level and mRNA level in active LN patients compared with healthy controls. We chose to study the role of IGFBP2 since it is a relatively understudied protein in the context of LN. We further validated that IGFBP2 significantly increased and correlated with SLE activity index in active LN patients. The receiver operator characteristic (ROC) curve suggested that plasma IGFBP2 had a high diagnostic efficiency for distinguishing between inactive and active LN patients (AUC = 0.992; 95% *CI* = 0.974–1.000; *P* < 0.001). We demonstrated neutralizing IGFBP2-downregulated CD4^+^ T cell activation, upregulated the ratio of Treg, downregulated AKT/mTOR/4E-BP1 pathway, and significantly improved nephritis in MRL/lpr mice. In all, our work demonstrated IGFBP2 as a biomarker specific for active LN and blocking IGFBP2 could be a new target for treating LN.

****Key messages**:**

- Plasma IGFBP2 is a promising diagnostic marker for distinguishing stable LN from active LN, and it is also a predictor for the poor prognosis of LN.
- Blockade of IGFBP2 can significantly improve the pathological damage of LN.
- IGFBP2 may regulate activation of CD4^+^ T and Treg ratio.
- Neutralizing IGFBP2 downregulates AKT/mTOR/4E-BP1 pathway.

**Supplementary Information:**

The online version contains supplementary material available at 10.1007/s00109-022-02241-z.

## Introduction

Systemic lupus erythematosus (SLE) is a chronic autoimmune disease characterized by inflammation in multiple organs [1]. Renal involvement is the primary cause of SLE morbidity and mortality, affecting approximately 60% of patients. Moreover, approximately a quarter of lupus nephritis (LN) patients die of end-stage renal disease (ESRD) [2]. Pathological changes associated with LN include glomerular and tubular edema, atrophy, interstitial inflammation, and/or fibrosis. Approximately 60% of LN patients and chronic renal disease symptoms manifest as interstitial inflammation or fibrosis [3]. In all, early intervention of renal symptoms is critical for the prognosis of SLE. Therefore, identification of new targets as biomarkers as well as treatments for LN is of great clinical value.

It has previously been reported that members of the insulin-like growth factor–binding protein (IGFBP) family may represent markers of lupus-induced renal damage. For example, the level of IGFBP4 is closely related to the chronic index of renal pathology [4]. The levels of plasma IGFBP2 in patients with LN were significantly increased, which may represent a new marker that reflects SLE disease activity [5]. IGFBP2 can be expressed in a variety of organs, tissues, and cells and exhibits complex functions as an intracellular or secreted protein. IGFBP2 is considered to be a factor involved in the inhibition of IGF activity and regulates the biological effects of IGF by controlling IGF distribution, function, and activity [6]. Increased circulating IGFBP2 may be a predictor of the longitudinal deterioration of renal function in a variety of kidney diseases, including acute kidney injury [7]. However, the role of IGFBP2 in LN and whether IGFBP2 can be a therapeutic target remain to be determined. We would measure the peripheral and renal IGFBP2 levels in patients with LN and examine the relationship between the levels and disease activity in this study. By administering neutralizing IGFBP2 Ab to MRL/lpr mice, we would also evaluate the effect of blocking IGFBP2 in vivo. In general, the study will define the role of IGFBP2 in LN diagnosis and prognosis and confirm if IGFBP2 could be a therapeutic target for treating LN.

## Materials and methods

### Subjects

A total of 57 patients with LN were recruited from the Affiliated Drum Tower Hospital of Nanjing University Medical School, and all patients fulfilled the American College of Rheumatology (ACR) revised criteria (1997) [8]. A total of 27 sex- and age-matched volunteers were recruited as healthy controls (HCs). LN inclusion criteria consisted of 18 years of age or older, The Systemic Lupus Erythematosus Disease Activity Index 2000 (SLEDAI-2 K) ≥ 8 combined with LN, and fulfilled the British Isles Lupus Assessment Group (BILAG) A 1 item or BILAG B grade 2 item [9]. The exclusion criteria consisted of patients with other connective tissue diseases complicated with an infection or tumor who were pregnant or breastfeeding.

The patients with LN were divided into two groups. SLEDAI-2 K was used to judge the level of disease activity [10]. SLEDAI-2 K scores ≤ 4 represented inactive disease, and SLEDAI-2 K scores ≥ 8 represented active disease. In this study, 16 patients with RA who fulfilled the 2010 ACR Rheumatoid Arthritis Classification Criteria [11] and 16 patients with Sjogren’s syndrome (SS) who fulfilled the 2002 American-European Consensus Group (AECG) Classification Criteria [12] were included as control groups. All patients exhibited active disease. The simple disease activity index (SDAI) of patients with RA was > 3.3 points, and patients with SS had damage to at least one system.

Venous blood and urine samples were harvested from all subjects. Peripheral blood mononuclear cells (PBMCs) were isolated by Ficoll density gradient centrifugation methods according to the manufacturer’s instructions (STEMCELL Technologies, Vancouver, CA). RNA was extracted from the PBMCs using TRIzol in accordance with the manufacturer’s instructions (Invitrogen, Carlsbad, USA).

### TMT-LC–MS/MS and RNA-seq

The proteomic profiling of nine patients with active LN using TMT-LC–MS/MS was analyzed on a Thermo Orbitrap Elite mass spectrometer (Thermo Finnigan, San Jose, CA). Differentially expressed (DE) proteins were identified and quantified. Total RNA was extracted from PBMCs and sequenced using Illumina HiSeq 2500 (Illumina, USA). Mass spectrometry and RNA-seq services were carried out by Shanghai Shengzi Biological Technology Co., Ltd.

The following selection criteria for the DE genes were used: FDR ≤ 0.05 and FC ≥ 2. DE genes were analyzed by DAVID 6.8 (https://david.ncifcrf.gov/) using the GO and Kyoto Encyclopedia of Genes and Genome (KEGG) databases. The protein sequence and human protein data from Ensembl were compared using BLASTP. A similarity of 85% was used as the cutoff to associate the protein with the transcriptome.

### Animals and treatment

C57BL6/J (B6) female mice (Animal Center of Nanjing Medical University, China) and MRL/lpr female mice (Shanghai SLAC Laboratory Animals Co., Ltd, Shanghai, China) were purchased at 4 weeks of age and housed in the Animal Center of Nanjing Drum Tower Hospital under specified pathogen-free (SPF) conditions. Sixteen-week-old MRL/lpr mice were randomly divided into three groups. The mice were intraperitoneally injected with PBS, 10 µg mouse IGFBP2 antibody (cat: MAB797, R&D), and rat IgG2A ISOtype control antibody (cat: MAB006, R&D) each week for 4 weeks.

### Real-time PCR

For real-time PCR, RNA was extracted from cells and tissues using TRIzol according to the manufacturer’s instructions (Invitrogen, Carlsbad, USA). cDNA was synthesized from 1 μg of total RNA using HiScript II Q RT SuperMix (Vazyme Biotech). RT–qPCR was performed in accordance with the manufacturer’s instructions for the SYBR Green Master MIX (Low ROX Premixed) and Applied Biosystems QuantStudio 6 Flex Real-Time PCR System. The primer sequences (Genscript Biotech, Nanjing, China) are listed in supplementary Table 1.

### Enzyme-linked immunosorbent assay (ELISA)

The concentrations of IGFBP2 in the plasma and supernatant of homogenized tissues were measured using an IGFBP2 R&D Systems ELISA kit (Minneapolis, MN; cat no: DBG200 for humans and cat no: DY797 for mice). All samples were diluted to 1:10 for plasma and 1:50 for the supernatants. The absorbance was read by spectrophotometry at 450 nm. A standard curve was constructed to calculate the concentration of IGFBP2. All measurements were repeated at different dilutions to confirm the validity of the analyses.

### Flow cytometry assay

The following reagents were used for flow cytometric analysis: mouse antibodies: CD3 BUV395, CD4 FITC, CD8 BV510, CD25 APC, CD69 Percp Cy5.5, Ki67 PE Cy7, Foxp3 PE, IFN-γ BV786, IL-4 PE-CF594, IL-17 BV421, IL-2 APC-R700, and live dye APC Cy7 (780). All antibodies were purchased from eBioscience (San Diego, CA). To detect intracellular factors, the cells were incubated with 0.1 μg/mL phorbol 12-myristate 13-acetate (PMA), 5 μg/mL ionomycin, and 25 μg/mL BFA at 37 °C for 6 h. All analytical flow cytometry was performed on a BD FACSCalibur^™^ Flow Cytometer (BD Immunocytometry Systems, San Diego, CA) using FlowJo software (TreeStar, Ashland, OR) for data analysis.

### Western blot analysis

SDS–PAGE and immunoblotting were used to detect the level of protein expression. Briefly, protein samples (20 µg) were resolved by 12% SDS–PAGE, electroblotted on nitrocellulose membranes (Bio-Rad), incubated overnight at 4 °C with the following antibodies: IGFBP2 (11065–3-AP, Proteintech), antibodies used for Western blotting, were bought from AiFang biological and listed as follows: p-RPS6KB1(AF14512), RPS6KB1(AF11049), p-4E-BP1(AF01102), 4E-BP1(AF03855), p-AKT(AF00453), AKT(AF01499), p-mTOR (AF00658), mTOR (AF02824). Then, the membranes were detected with HRP-linked secondary antibodies. GAPDH (cell signaling) was used as an internal reference. The protein bands were visualized using a Tanon-5200 Imaging System (Shanghai Tanon Science & Technology).

### Assessment of kidney injury

LN was pathologically classified according to the International Society of Nephrology/Renal Pathology Society [13]. Kidney biopsy samples from 15 LN patients, 3 primary nephrotic syndrome patients, and normal renal tissue samples adjacent to renal cancer were stained with hematoxylin and eosin (H&E), periodic acid-Schiff (PAS), and Masson’s trichrome stain. The cells were counted under high-power magnification to calculate the histological score for kidney disease [14, 15]. Antibodies and dyes used for IHC and IF were listed as follows: anti-IGFBP2 (ab188200, Abcam), Alexa 488-conjugated goat anti-rabbit IgG, Alexa Fluor 594-conjugated goat anti-rabbit IgG (Thermo Fisher Scientific), and Hoechst 33258. The sections were observed under an inverted fluorescence microscope (Olympus, Tokyo Japan). ImageJ software was used for image acquisition and processing. The level of mouse urine protein was determined using a Bradford assay kit (KGI, Nanjing, China).

### Statistical analysis

Statistical analyses were performed using GraphPad Prism version 7.0 for Windows (GraphPad Software, La Jolla, CA). All values were expressed as the mean ± SD. DE proteins were identified by the fold-change threshold (fold ≥ 1.5) and an independent *t* test. The data at the time points before and after treatment were tested using a paired *t* test, and comparisons between the other two groups were assessed using an unpaired *t* test. Three groups or more than three groups were compared using one-way analysis of variance. A value of *P* < 0.05 was considered to be statistically significant.

## Results

### Differential proteins are detected and analyzed between the active LN and HC groups

**Fig. 1:**
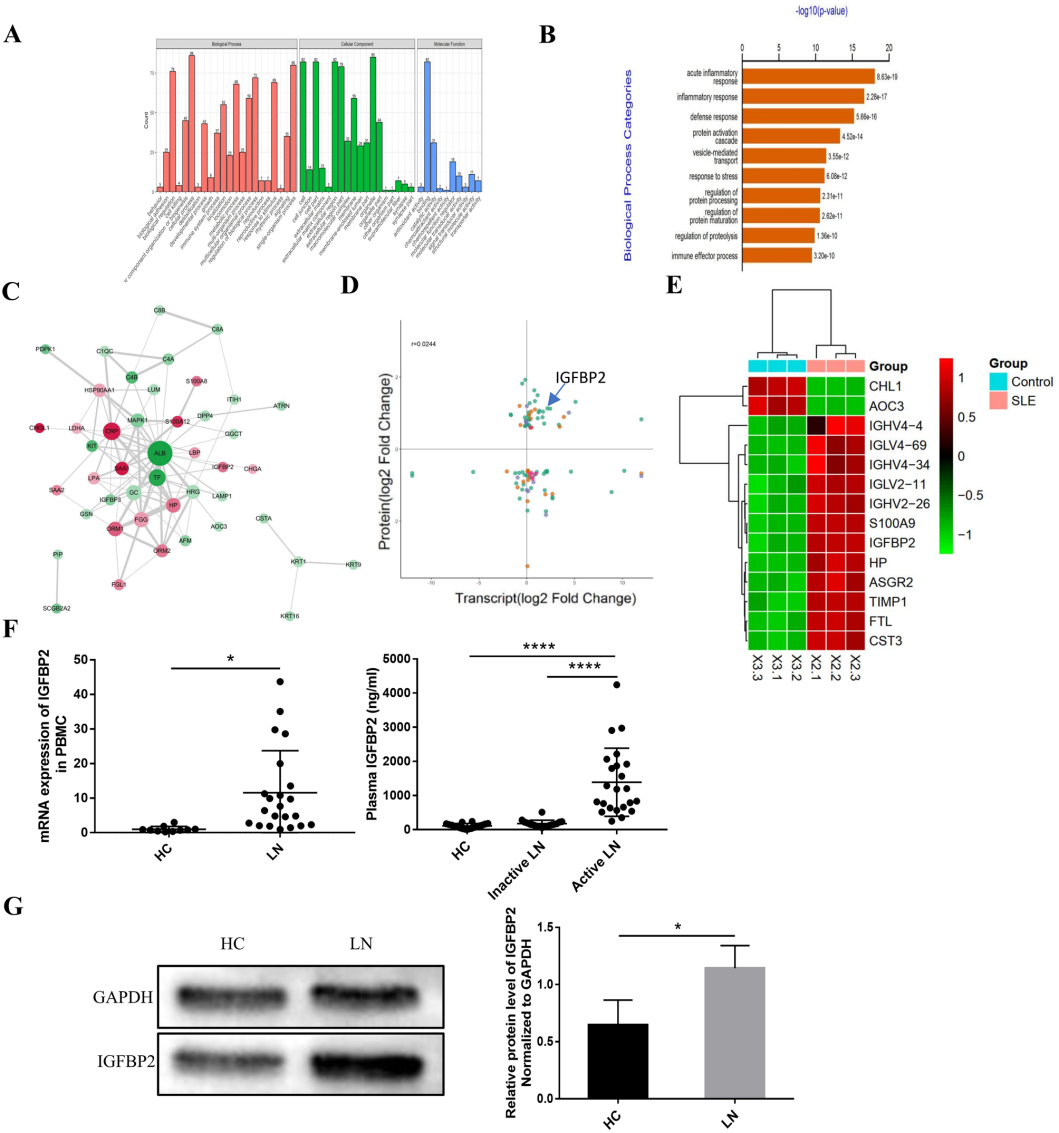
TMT detection of differential protein expression between the active LN and HC groups. **A** Statistical histograms of GO ontology biological processes, cellular components, and molecular functions. **B** Bar graph displaying the top 10 enriched GO biological process functions. **C** Network analysis diagram. **D** Scatter plot of DE proteins and corresponding transcripts analyzed by the proteome and transcriptome. The *x*-axis is the transcript log 2 (fold-change), and the *y*-axis is the protein log 2 (fold-change). *R* = 0.0244 was the Pearson correlation coefficient of the two sets of data. The transcript was differentially expressed according to whether it was expressed in different colors. **E** Heatmap of proteins that were differentially expressed at the proteome and transcriptome levels and were consistently up- or downregulated. **F** Real-time PCR validation tests of IGFBP2 gene expression were performed in peripheral blood mononuclear cells (PBMCs) of the control (*n* = 10) and LN (*n* = 24) groups. ELISA validation tests were performed to verify IGFBP2 expression in the plasma of the controls (*n* = 18), inactive LN (*n* = 18), and active LN patients (*n* = 23). **G** Validation of IGFBP2 expression in the PBMCs of HC and LN patients by WB (*n* = 3). *P < 0.05; **P < 0.01; ***P < 0.001; ****P < 0.0001

To compare differential protein expression between the active LN and HC groups, the plasma of 18 patients with active LN and 9 healthy controls (HCs) was sequenced. Baseline data of the enrolled LN patients are listed in Table 1. The GO and KEGG databases in DAVID 6.8 (https://david.ncifcrf.gov/) were used to explore the potential role of differential expression (DE) proteomics (Fig. 1A, B). The results showed that the most significant biological process enrichment was the regulation of acute inflammatory response, inflammatory response, defense response, and protein activation. The protein interaction network analysis diagram showed the interaction between multiple proteins (Fig. 1C). All DE proteins and their associated transcripts in both the proteomic and transcriptomic were analyzed and showed a total of 97 differentially expressed proteins, including 35 upregulated proteins and 15 downregulated proteins (Fig. 1D). Heatmap of 14 genes that are top differentially expressed is listed in Fig. 1E.

**Table 1:** Baseline data of enrolled LN patients (*n* = 57)

**Variable**	**SLE patients with active renal diseases (*n* = 38)**	**SLE patients with inactive renal diseases (*n* = 19)**	***P*-value**
Females/males	34/4	17/2	1.000
Age, years(range)	29.5 [24.8–41.5]	40 [27.0–48.0]	0.109
Disease duration, years(range)	3 [0.5–7.0]	5 [2.0–10.0]	0.129
Clinical characteristics			
Malar rash (%)	18(47)	2(10)	**0.006**
Mouth ulcers (%)	12(31)	2(10)	0.173
Arthritis (%)	9(23)	1(5)	0.176
Serositis (%)	13(34)	1(5)	**0.039**
CNS disease (%)	2(5)	0	0.548
Hematological disease (%)	8(21)	4(21)	1.000
Lung disease (%)	12(31)	0	**0.003**
ANA positivity (%)	38(100)	15(79)	**0.017**
Anti-dsDNA positivity (%)	30(79)	8(42)	**0.005**
Low C3 or C4 (%)	36(94)	10(52)	**0.001**
SLEDAI-2 K score (range)	14 [11.75–18.00]	2 [2.00–3.00]	**< 0.001**
Global BILAG-BR score (range)	22 [19.75–24.00]	12 [11.00–13.00]	**< 0.001**
Medications			
Hydrochloroquine (%)	32(84)	15(79)	0.902
Azathioprine (%)	5(13)	2(10)	1.000
Cyclophosphamide (%)	9(24)	2(10)	0.406
Leflunomide (%)	6(16)	3(16)	1.000
Mycophenolate mofetil (%)	12(32)	4(21)	0.404
Steroids (%)	33(86)	16(84)	1.000

Continuous variables were expressed as P50 (P25, P75). Mann–Whitney *U* tests were used for continuous variables. Enumeration data were shown as *n* (%), and analyzed through the chi-square test. Significant *P*-values marked in bold

### IGFBP2 is validated to be significantly high in plasma from active LN patients

IGFBP2 was selected as the target protein, and larger samples were selected to further validate the proteome and transcriptome results. The level of plasma IGFBP2 in active LN patients (*n* = 23) was significantly higher than that of the HC group (*n* = 18) and inactive LN (*n* = 18) (*P* < 0.01), whereas there were no significant changes observed compared with the HC group in inactive LN patients, as shown in Fig. 1F. The level of IGFBP2 mRNA and protein expression in the PBMCs of active LN patients was higher than that in the HC group (*P* < 0.05) (Fig. 1F, G).

**Fig. 2:**
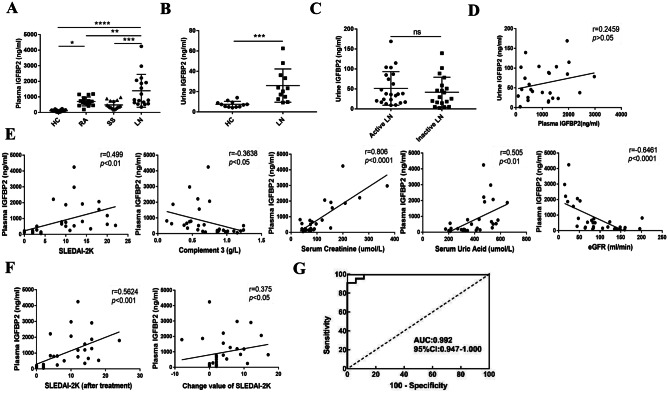
The level of plasma IGFBP2 expression in LNs and correlation analysis with clinical indicators. **A** Level of IGFBP2 expression in the plasma of RA (*n* = 16), SS (*n* = 16), active LN (*n* = 17), and control (*n* = 18) patients. **B** The level of urine IGFBP2 expression in LN patients (*n* = 12) was higheer than that in HC (*n* = 12) patients (*P* < 0.01). **C** There was no significant difference between urinary IGFBP2 in patients with inactive (*n* = 19) and active SLE (*n* = 23). **D** There was no correlation between plasma and urinary IGFBP2 levels in LN patients (*n* = 26). **E** Correlation analysis between plasma IGFBP2 levels and SLE activity-related indicators. **F** Correlation analysis between the plasma IGFBP2 and SLEDAI score at 3 months after treatment and change in SLEDAI before and after treatment. **G** The ROC curve revealed that IGFBP2 showed an AUC value of 0.992, with a sensitivity of 0.909 and specificity of 1.0 (95% *CI* 0.974–1.0, *P* < 0.001) at the cutoff of 512 ng/mL between inactive and active LN. *P < 0.05; **P < 0.01; ***P < 0.001; ****P < 0.0001. *ns*, not statistically significant

We also detected the levels of plasma IGFBP2 in other connective tissue diseases (e.g., RA and SS). The results showed that the expression of IGFBP2 was slightly higher in patients with SS (*n* = 16, *P* > 0.05) and higher in patients with RA (*n* = 16, *P* < 0.05) than that in HCs (*n* = 18), whereas the level of IGFBP2 in active LN patients (*n* = 17) was significantly higher than that in RA and SS patients (*P* < 0.01) (Fig. 2A). In addition, we found similar results with detecting urine IGFBP2 expression level in LN patients. However, there was no statistically significant clinical correlation in IGFBP2 between the plasma and urine (Fig. 2B, C, D), suggesting that the level of urine IGFBP2 could not reflect the activity of renal damage.

### High IGFBP2 expression can predict the severity and prognosis of LN patients

Correlation of IGFBP2 with clinical indicators was further analyzed. The results showed that the expression of IGFBP2 was significantly positively correlated with the SLEDAI-2 K, serum creatinine, and uric acid (*P* < 0.01) and was negatively correlated with complement 3 (*P* < 0.05) and estimated glomerular filtration rate (eGFR) (*P* < 0.01) (Fig. 2E). There was a significant positive correlation between the expression of IGFBP2 and the SLEDAI-2 K score after 3 months of treatment in active LN patients (*r* = 0.562; *P* < 0.01), and a significant positive correlation between the change in SLEDAI-2 K before and after treatment (*r* = 0.375; *P* < 0.05) (Fig. 2F), suggesting that plasma IGFBP2 could be used as an indicator of poor disease prognosis. The diagnostic ability of IGFBP2 to predict the severity of SLE was evaluated by receiver operating characteristic (ROC) curve. The results showed that the area under the curve (AUC) of IGFBP2 was 0.992 (95% *CI*: 0.974–1.000); *P* < 0.001 (Fig. 2G), with a sensitivity of 90.9% and specificity of 100% at a cutoff of 512 ng/mL at the greatest Youden index of 0.909.

### IGFBP2 expression is elevated in the renal tubules of LN patients

**Fig. 3:**
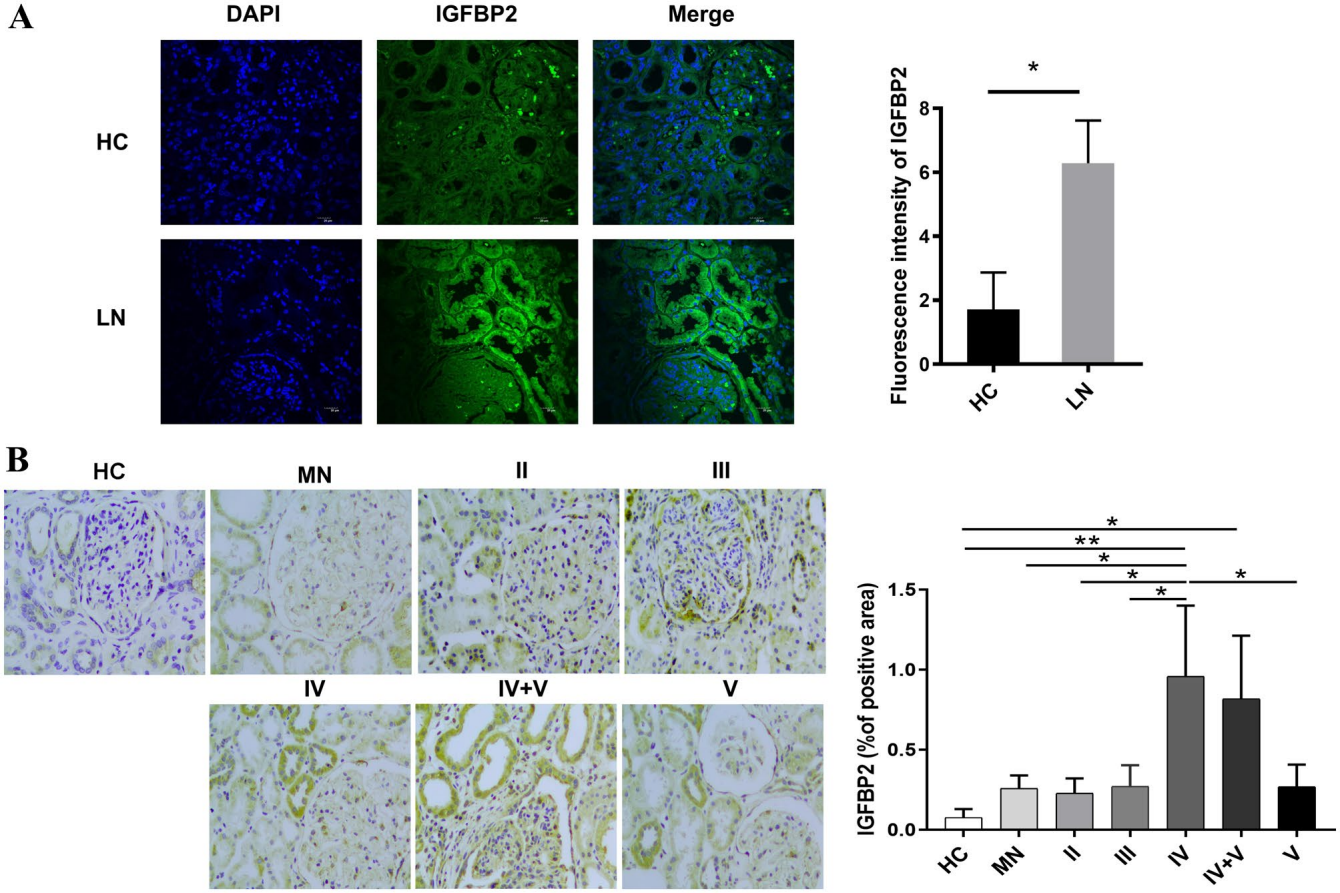
The level of IGFBP2 expression in the kidneys of LN. **A** IGFBP2 expression in normal kidney issues and LNs by IF staining (*n* = 3). **B** IHC staining of IGFBP2 in HC (*n* = 3), MN (*n* = 3), and II–V type LN kidneys (*n* = 3). Original magnification, × 400. The positive area is quantitatively displayed as a histogram on the right. *P < 0.05; **P < 0.01

The levels of IGFBP2 expression in kidneys of cases with membranous nephropathy (MN) and type II–V LN were examined by immunohistochemistry (IHC) and immunofluorescence staining (IF). Compared with HCs, the kidneys of LN patients exhibited increased IGFBP2 deposition and were primarily located in the renal tubules and glomerular podocyte distribution regions (Fig. 3A) by IF staining. The results of IHC staining showed that obvious deposition of IGFBP2 was observed in the renal tubules of LN patients, especially in class IV and IV + V patients (*P* < 0.05), as shown in Fig. 3B. But it still remains unclear whether IGFBP2 deposition in the local renal tubule is involved in the damage of lupus renal tubular cells and aggravates renal interstitial inflammation.

### High expression of IGFBP2 in MRL/lpr mice

**Fig. 4:**
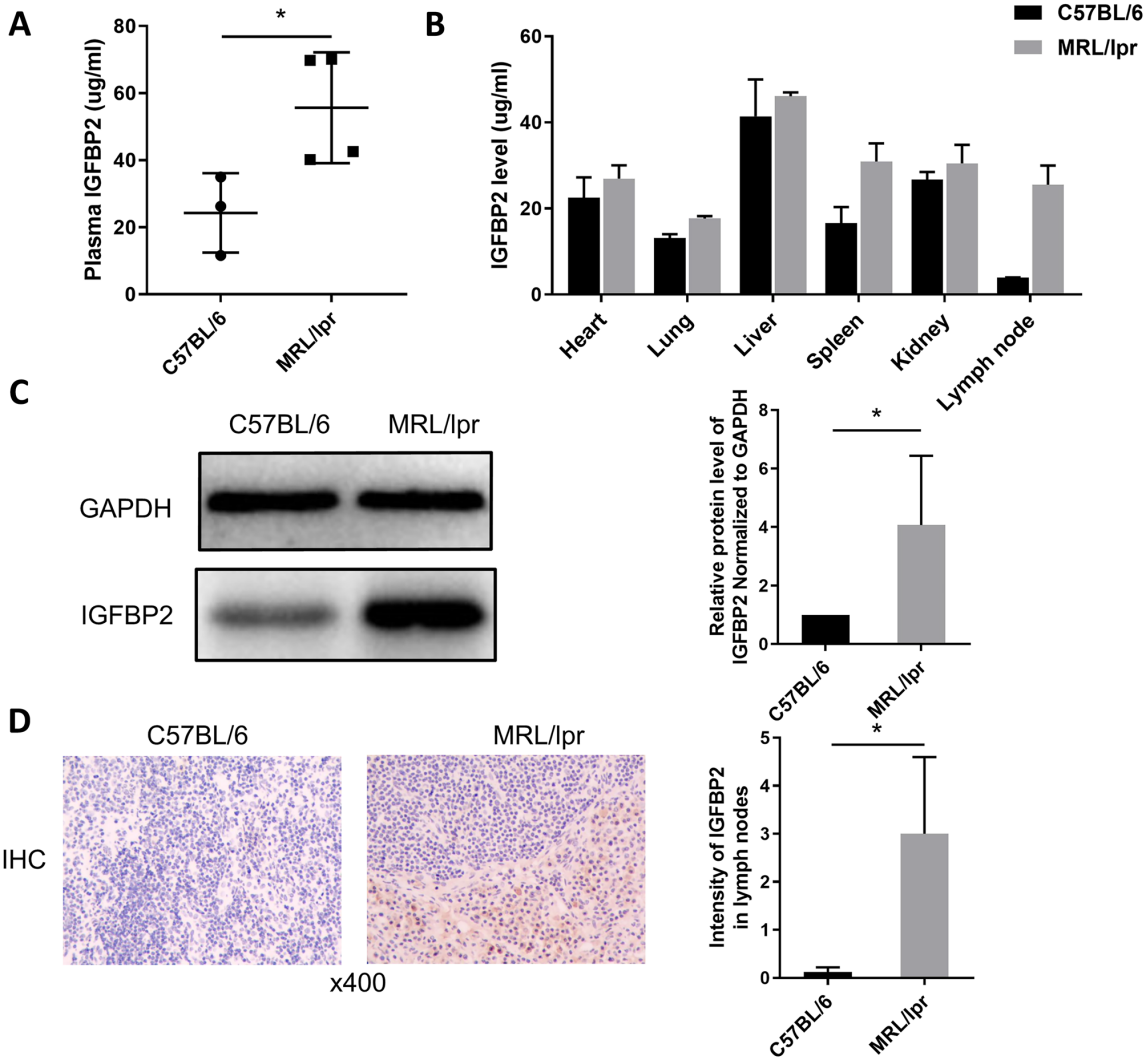
The distribution of IGFBP2 in the organs of mice. **A** The level of IGFBP2 expression in the blood of MRL mice (16 weeks) (*n* = 4) was higher than that of C57BL/6 mice (*n* = 3). **B** ELISA detection of the IGFBP2 content in the various organs of C57BL/6 and MRL/lpr mice revealed that in most organs of MRL/lpr mice, the protein content of IGFBP2 was higher than that of the C57BL/6 mice. **C** WB showed the expression of IGFBP2 in the lymph nodes of MRL/lpr and C57BL/6 mice (*n* = 3). **D** IHC showed that the level of IGFBP2 in the lymph nodes of MRL/lpr was significantly higher than that in the lymph nodes of C57BL/6 mice (intracellular tan deposition) (*n* = 3). *P < 0.05

Studies have shown that IGFBP2, as a secreted protein, can be expressed in multiple organs. To explore the distribution of IGFBP2, we selected age-matched MRL/lpr (16 W) and C57BL/6 (B6) mice to detect the level of IGFBP2 expression in different organs. The level of plasma IGFBP2 in MRL/lpr mice was higher than that in C57BL/6 mice (*P* < 0.05) (Fig. 4A). Moreover, the expression of IGFBP2 in most organs of the MRL/lpr mice was higher than that in C57BL/6 mice, especially in the lymph nodes detected by ELISA and WB (Fig. 4B, C). As shown in the IHC pathology of lymph nodes, a large amount of IGFBP2 (tan substance) was deposited in the cytoplasm of T cells between the follicles (Fig. 4D), indicating that the significantly increased level of IGFBP2 in the plasma and organ tissues may be involved in the pathogenesis of SLE.

### IGFBP2 blockade improves LN in MRL/lpr mice

**Fig. 5:**
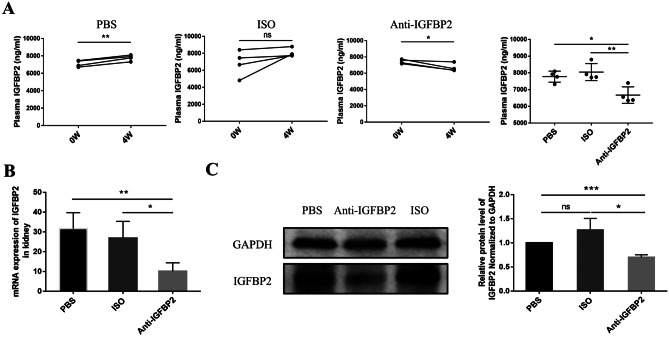
The IGFBP2 blocking effect in mice following treatment. **A** MRL/lpr mice were divided into the PBS group (*n* = 4), ISO group (*n* = 4), and anti-IGFBP2 group (n = 4). The ELISA results showed that compared with PBS and ISO groups, the level of plasma IGFBP2 in the anti-IGFBP2 group was decreased. **B** PCR and **C** WB showed the level of renal IGFBP2 protein expression in each of the three groups. *P < 0.05; **P < 0.01; ***P < 0.001. *ns*, not statistically significant

To further verify our findings, we used an in vivo blockade of IGFBP2 to observe the therapeutic effect and immune regulation of T cell in LN. We found that the level of plasma IGFBP2 in the PBS group was significantly increased after 4 weeks, suggesting that IGFBP2 expression increases with LN progression. The level of plasma IGFBP2 in the anti-IGFBP2 group was lower than that prior to treatment and was lower than that of the other two groups (*P* < 0.01) (Fig. 5A), and that the renal expression of IGFBP2 was significantly decreased (*P* < 0.05) (Fig. 5B, C). Therefore, the treatment of anti-IGFBP2 antibody can reduce the level of IGFBP2 in the plasma and renal tissues in MRL/lpr mice, achieving the goal of blocking IGFBP2 in vivo.

**Fig. 6:**
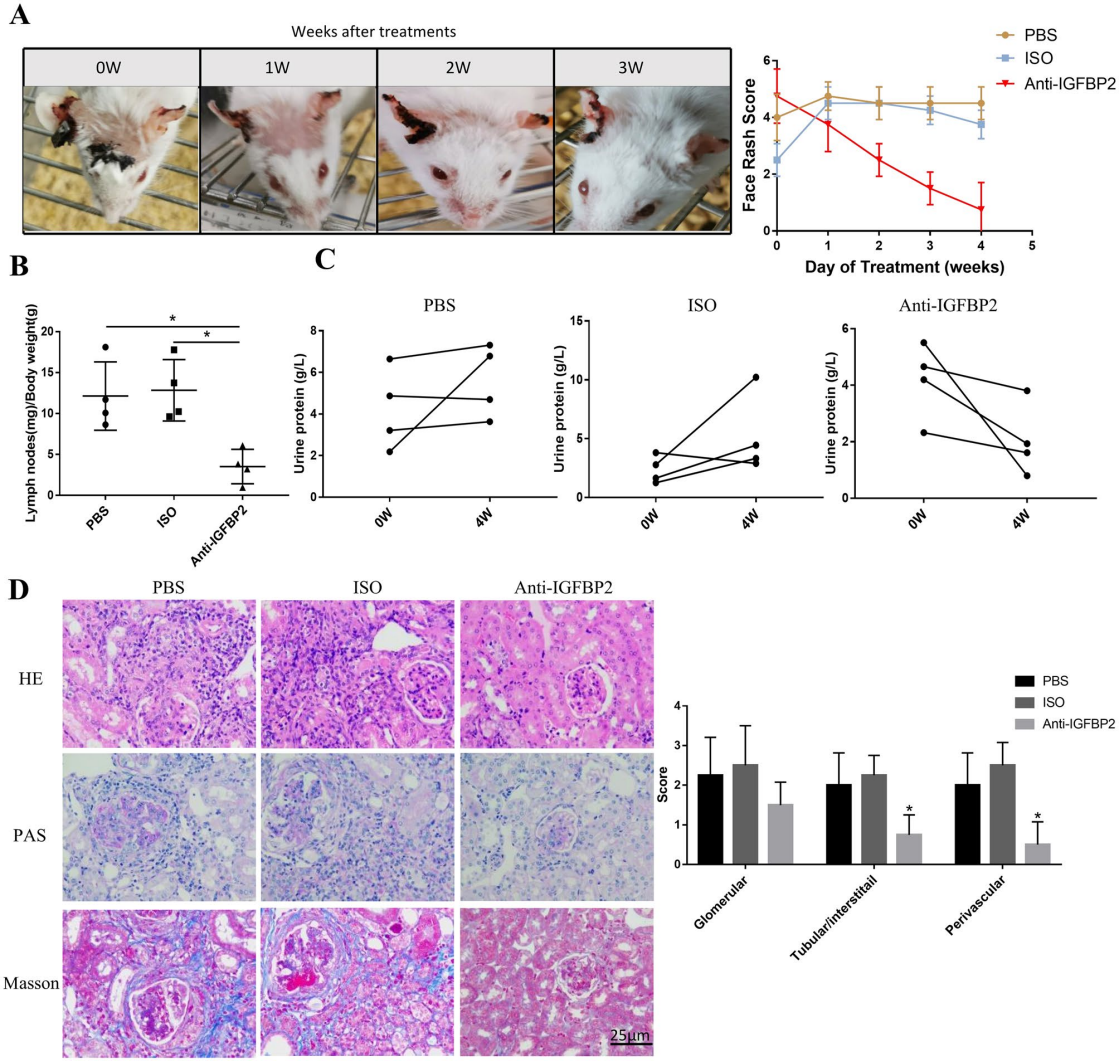
Improvement of LN after a IGFBP2 blockade. **A** MRL/lpr mice were divided into the PBS group (*n* = 4), ISO group (*n* = 4), and anti-IGFBP2 group (*n* = 4). The skin lesions in the anti-IGFBP2 group were significantly improved. **B** Compared with the PBS and ISO groups, the lymph/body weight ratio was decreased in the anti-IGFBP2 group. **C** Trend in the reduction of urine protein following treatment. **D** HE, PAS, and Masson staining (original magnification, × 400), quantified histogram of glomerulus, tubulointerstitium and perivascular pathology. *P < 0.05; **P < 0.01

The skin lesions in MRL/lpr mice after neutralizing IGFBP2 were significantly improved (Fig. 6A), the weight of peripheral lymph nodes were substantially reduced, and the lymph/body weight ratio was decreased (Fig. 6B). Compared with the PBS and ISO groups, there was a downward trend in urine protein in the anti-IGFBP2 group (*P* > 0.05) (Fig. 6C). Kidney histopathology showed that IGFBP2 blockade significantly decreased glomerular cell proliferation, mesangial matrix deposition, and the histologic score. Moreover, the severities of renal tubulointerstitial and perivascular lesions were improved (Fig. 6D).

### Blockade of IGFBP2 inhibits the activation of CD4^+^ T cells and AKT/mTOR/4E-BP1 pathway

**Fig. 7:**
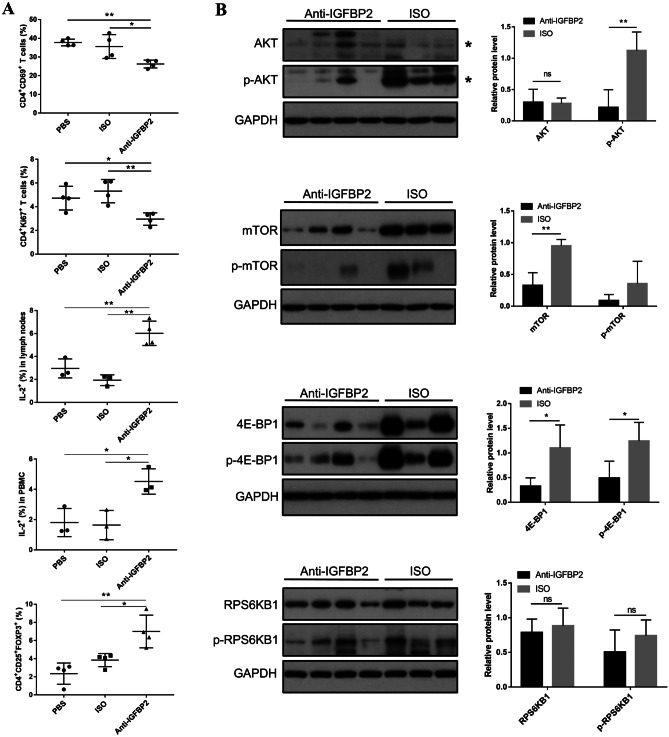
IGFBP2 blockade affects T cells and AKT/mTOR pathway. **A** Effect on peripheral blood T cell immunity by flow cytometry in PBS group (*n* = 4), ISO group (*n* = 4), and anti-IGFBP2 group (*n* = 4). **B** Protein expression and phosphorylation levels of AKT, mTOR, RPS6KB1, and 4E-BPI in anti-IGFBP2 group (*n* = 4) and ISO group (*n* = 3) were detected by Western blotting. The quantitative analysis of proteins was performed using ImageJ software. *P < 0.05; **P < 0.01; *ns*, not statistically significant

PBMC and lymph nodes of mice were collected and analyzed by flow cytometry; the results showed that IL-2 secretion was obviously increased in anti-IGFBP2 group. And, a blockade of IGFBP2 elevated the ratio of Tregs inhibits the proliferation and activation of CD4^+^ T cells, thereby achieving the possibility of inhibiting the progression of lupus (Fig. 7A) (*P* < 0.05).

There is significant evidence that mTOR activity is increased in SLE [16]. According to the above results, mTOR pathway regulates the maintenance of immune tolerance at the level of the regulatory T cell. Moreover, the mTOR signaling pathway is regulated by IGFBP2 in cancer; we next examined the effect on AKT/mTOR signaling pathway with IGFBP2 blockade in mouse models of lupus. As shown in Fig. 7B, compared with ISO group, the level of mTOR and the phosphorylation of AKT and mTOR were significantly downregulated in anti-IGFBP2 group (*P* < 0.05). Signaling by the PI3K/Akt/mTOR pathway profoundly affects mRNA translation through phosphorylation of downstream targets such as 4E-BP and RPS6KB1 [17]; we further explored the level of phosphorylation of 4E-BP1 and RPS6KB1 in lymphocytes of mouse models of lupus with IGFBP2 blockade treatment. The results showed that IGFBP2 blockade mediated the inhibition of mTOR phosphorylation which led to decreased level of phosphorylation of 4E-BP1, while there was no significant changes in protein and phosphorylation of RPS6KB1. Collectively, all the data showed that IGFBP2 was involved in the progression of LN by activating the mTOR/AKT/4E-BP1 signaling pathway. Treatment with the IGFBP2-neutralizing antibody may be an effective therapeutic strategy for LN.

## Discussion

LN may cause clinical manifestations like glomerulonephritis or nephrotic syndrome, which negatively impacts the prognosis of SLE [18, 19]. The traditional renal injury markers (e.g., serum creatinine and blood urea nitrogen) are not sensitive enough to diagnose early renal injury. Our results showed that blockade of IGFBP2 could significantly upregulate IL-2 secretion and Treg ratio and inhibit the proliferation and activation of CD4^+ ^T cells. The study suggested that plasma IGFBP2 level has an extremely high diagnostic efficiency and can serve as an indicator of disease prognosis.

IGFBP2 has been discovered as an independent prospective biomarker for the early diagnosis of AKI [20], but there has been few research on its relationship to the progress of SLE. Our results demonstrated that IGFBP2 was significantly higher in LN patients than in SLE patients without renal involvement. Hence, IGFBP2 can distinguish active renal SLE from active nonrenal or inactive SLE. In the kidneys of LNs, our study indicated that IGFBP2 expression was enhanced and that IGFBP2 was largely deposited in the renal tubules and glomerular podocyte areas. We hypothesize that a significant buildup of IGFBP2 in the nearby renal tubules may exacerbate renal interstitial injury. In mouse model, the process of LN was improved by neutralizing IGFBP2; our findings suggest that a blockade of IGFBP2 may be a potential target for the clinical treatment of LN.

Studies showed that PBMCs could secrete a variety of IGFBPs, and the level of IGFBP2 mRNA expression in T cells was higher than that in B cells [21]. It has previously been reported that stimulation and activation with PMA or anti-CD3 antibodies can increase the IGFBP2 mRNA and intracellular IGFBP2 expression in T cells, which indicates the involvement of IGFBP2 in T cell activation and proliferation [22]. In our study, we found that IGFBP2 blockade downregulated the proliferation and activation of CD4^+ ^T cells and upregulated the ratio of Treg. These results suggest that IGFBP2 play an essential role in SLE pathogenies. Furthermore, we recognized IGFBP2 blockade suppressed the activation of ATK/mTOR pathway in lymphocytes of MRL/lpr mice. AKT/mTOR pathway is essential for the proper activation and proliferation of T cells in autoimmunity [23]. mTOR signaling pathway also can preferentially inhibit the differentiation of Tregs and increase T-cell proliferation [24]. Serving as the downstream targets of mTOR, 4E-BP1 and eIF4E further mediated the translation initiation. And, mTOR pathway is activated in T cells in parenchymal organs in SLE patients and lupus prone mice [25]. Our results suggest that IGFBP2 may regulate activation of CD4^+ ^T and Treg differentiation via AKT/mTOR pathway.

IGFBP2 can bind to IGF ligands and display IGF-dependent growth inhibitory effects on many cell types; it also acts independently of IGFs by putative mechanisms such as direct integrin binding (RGD domains) or possibly specific receptors [26]. We have not studied further to investigate whether IGFBP2 affected T cell immune regulation via IGF. IL-2 is a cytokine that reflects Treg cell function, homeostasis, and survival and thus is key to the improvement of LN [27, 28]. We also detected the change of IL-2 level was associated with IGFBP2, while the mechanism has been unknown in our study. Nephritis, which is a kidney disorder, is a complication of systemic lupus erythematosus. And, IGFBP2 is closely related with kidney injury. More experiments are needed to reveal the in-depth pathogenic mechanism of IGFBP2 in nephritis and its regulatory effect on other immune cells. Currently, administering the IGFBP2-neutralizing antibody to block IGFBP2 cannot be used in clinical practice. More clinical trials are needed to optimize this method.

## Conclusions

Our results demonstrate that plasma IGFBP2 may represent a new biomarker for LN. Thus, blocking IGFBP2 may provide a potential biological target for the treatment of LN.

## Supplementary Information

Below is the link to the electronic supplementary material.

Supplementary file1 (RAR 4405 KB)

Supplementary file2 (RAR 56 KB)

Supplementary file3 (RAR 24223 KB)

Supplementary file4 (RAR 20237 KB)

Supplementary file5 (RAR 3755 KB)

Supplementary file6 (RAR 19 KB)

Supplementary file7 (RAR 664 KB)

Supplementary file8 (TIF 326 KB)

## Data Availability

All data are included in the manuscript or are available from the corresponding author. The raw sequence data reported in this paper have been deposited in the Genome Sequence Archive (Genomics, Proteomics & Bioinformatics 2021) in National Genomics Data Center (Nucleic Acids Res 2022), China National Center for Bioinformation/Beijing Institute of Genomics, Chinese Academy of Sciences (GSA-Human: HRA002529) that are publicly accessible at https://ngdc.cncb.ac.cn/gsa-human. The proteome data reported in this paper have been deposited in the OMIX, China National Center for Bioinformation/Beijing Institute of Genomics, Chinese Academy of Sciences (https://ngdc.cncb.ac.cn/omix: accession no. OMIX001227).
