# Supplementary figures and images for: IGFBP2 function as a novel biomarker for active lupus nephritis
Source: J Mol Med (Berl). 2022 Aug 25;100(10):1479–91. doi: 10.1007/s00109-022-02241-z (PMC9470718; doi:10.1007/s00109-022-02241-z)

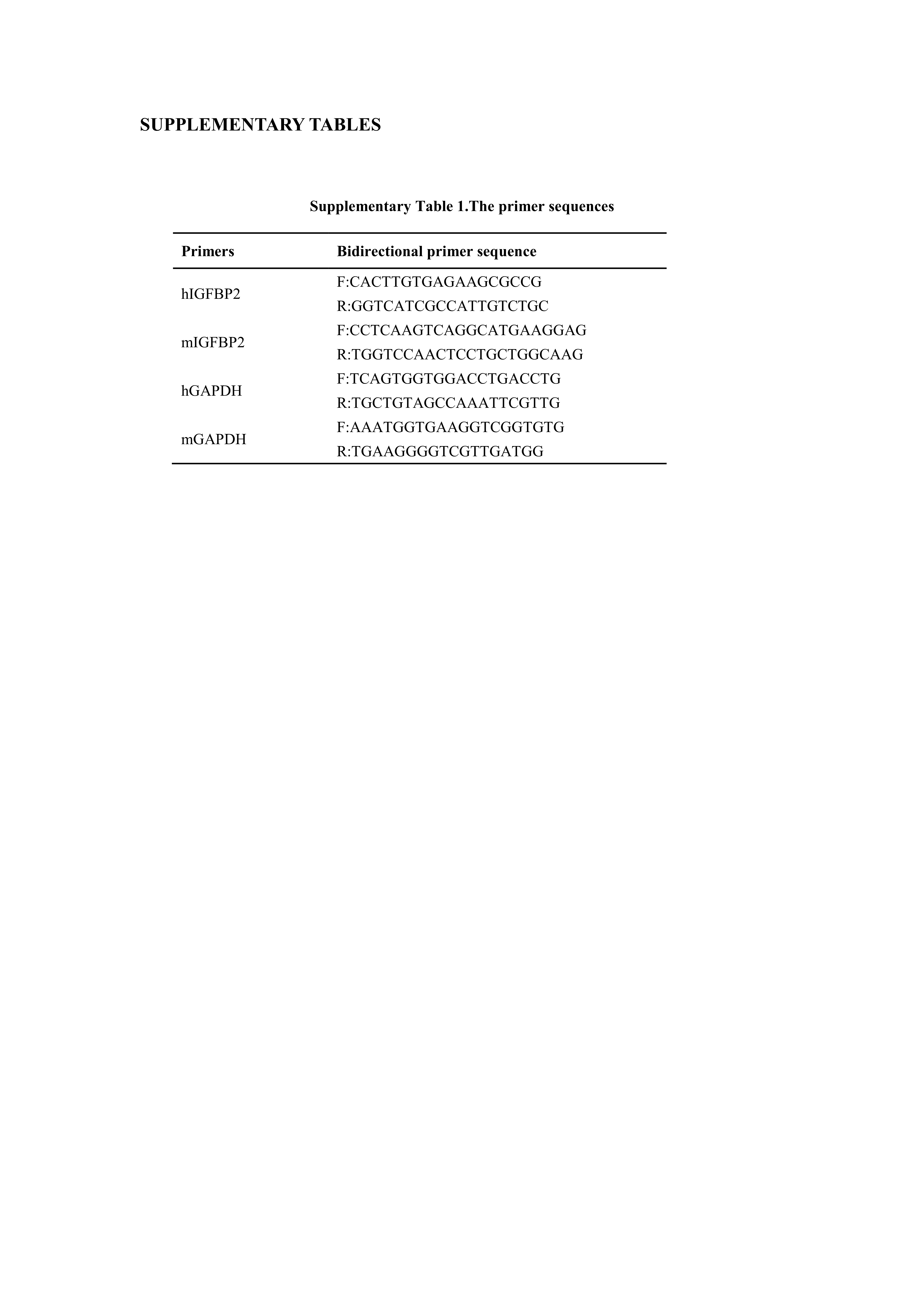

Supplement: Supplementary file 8 — Supplementary file8 (TIF 326 KB) [file 109_2022_2241_MOESM8_ESM.tif]
